# Speaking out for speakers: a guide for and analysis of robot speaker design

**DOI:** 10.3389/frobt.2024.1394700

**Published:** 2024-11-29

**Authors:** Nnamdi Nwagwu, Adeline Schneider, Tyler K. Phelps, Brian J. Zhang, Naomi T. Fitter

**Affiliations:** Collaborative Robotics and Intelligent Systems Institute (CoRIS), Oregon State University, Corvallis, OR, United States

**Keywords:** robot sound, speakers, speaker design, human-robot interaction, speaker analysis

## Abstract

Despite sound being a promising modality of communication in robotics (possessing, for example, the ability to improve people’s perceptions of robots and help localize robotic systems in space), its facilitator, speakers, are a seldom-explored topic of study in robotics literature. To address this gap, we conducted three explorations into physical speaker design that identified what current robot speakers lack and potential remedies, low-level design improvements, and *post hoc* hardware additions. Further, we detail and explore the application of speakers on three different robotic platforms (including one industrial robot used for construction), the last evaluation of which involved an empirical study (N=21) that sought to better understand the implications associated with poor-quality speakers in a mock service robotics context. Our results suggest that greater internal cavity volume is a key strength in speaker design. We also observed greater effects of the presence (vs. absence) of a service robot voice compared to other factors. This work can inform the process of creating custom speakers for robots and augmenting current robotic systems with new speaker additions (whether commercial or custom, and across use contexts from construction to service). In particular, the work can help to guide roboticists who may be unfamiliar with nuanced audio engineering techniques and designers who seek to improve robotics platform standards with human interlocutors in mind.

## 1 Introduction

Speaker quality is an important facet of the characterization of technology. For example, sound profiles emergent from electric car speakers can influence how annoying, aggressive, and powerful the car seems to potential users (Lennström et al., 2011), in addition to potentially enhancing and differentiating a car’s branding (Wagner et al., 2017). Sounds that naturally emerge from a vehicle, such as HVAC noises, can also have important implications on user favor and experience (Yoon et al., 2012; Leite et al., 2009). Examples and insights from vehicle sound can help to inform and guide the more recent development of robot sound as a research area. With inspiration from the adjacent vehicle sound space, our own past work demonstrated that robot sound influences people’s social perceptions of robots (Zhang et al., 2021) and monetary value judgements for these types of systems (Zhang et al., 2022). Between the lively work in neighboring fields like electric car sound and the insights gained so far on robot sound, there is good support of the importance of paying attention to how robots sound.

Despite the evidence of robot sound’s impacts from the work of our team and others (e.g., (Pelikan and Jung, 2023; Robinson et al., 2021; Zhang et al., 2021)), studies of robot speakers are exceedingly rare. The only existing study about speaker properties in human-robot interaction (HRI) looks at the effects of spatial audio on interactions with a humanoid robot (Robinson et al., 2023). This interaction is far from the only expected scenario of interest in which speakers might have an impact. For example, consider the effects that different frequency content (a characteristic resultant from lower-level speaker properties) can have on perceived pitch, tone, or intonation.

We know that these sound components are important to speech in particular. Affective information in the voice plays a key role in forming initial impressions (Lavan and McGettigan, 2023). Likewise, voice-based robot sound influences people’s expectations and attributions of robot characteristics. For example, people attribute robots with more human-like and smoother physical features with more natural (or human-like) voices (McGinn and Torre, 2019). Apparent robot voice gender also has affected credibility, trustworthiness, and engagement of the opposite sex in HRI studies (Siegel et al., 2009; Song et al., 2020). Similarly, expressivity of voice or modulating intonation increased engagement with children in a reading task (Kory Westlund et al., 2017). The ability to capably present the range and quality of audio needed to (as just one example) produce the right style of speech for a given situation depends on currently unspoken qualities and expectations of robot speakers. Thus, the robot speaker research gap has important implications on human perceptions of robotic systems.

*The central goal of this research is to establish a better knowledge base surrounding a seldom-studied element of robots (i.e., speakers) and to offer beginning speaker design and selection guidelines for robots, with an emphasis on service robots*, but including cases such as industrial robots in construction. Section 2 provides key background information on speakers, especially for those who may not have considered them before. Section 3 evaluates the speakers of two robots (i.e., the Stretch RE2 and Quori service robots), along with commercial and custom speaker options, to establish reference points and guidelines for robot speaker selection and integration. To highlight potential advantages of custom speaker use in particular, Section 4 provides case examples for how custom speakers can be tailored to specific applications in which commercial speaker use may be infeasible, such as the harsh environments of the construction industry. Section 5 provides an empirical evaluation of the impacts of speaker quality in a mock service robot context. Lastly, Section 6 discusses the main takeaways and conclusions of the presented work.

## 2 Background on speakers and speaker design

### 2.1 Evaluation criteria

**FIGURE 1:**
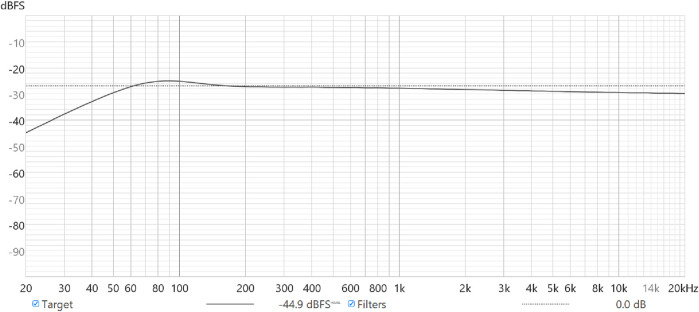
Plot of the Harman target for a bass-limited speaker (solid line) compared to a completely flat frequency response (dotted line) for reference.

The determination of what qualifies as a good speaker is often a more nuanced than common knowledge would suggest. There is no one clear definition of “good” speaker quality, but a speaker’s physical properties (such as frequency response curves) can help to frame relative advantages and disadvantages. A frequency response determines the relative loudness of all frequencies played back by loudspeakers (a common synonym of “speaker”). User preference often varies based on the frequency responses of speaker equipment. For example, some literature finds that flatter responses at lower frequencies, with smaller step-based increases in decibels (dB) thereafter, are most preferred in listening tests (Gabrielsson et al., 1988). Additionally, perceptual qualities, such as clarity, spaciousness, brightness, softness, nearness, loudness, and fidelity have been linked to changing decibel levels of frequency bands (Gabrielsson et al., 1990). These factors, however, are not necessarily direct determinants of the binary association of good vs. bad quality. Models from listening studies by Harman International Industries bridge the gap in determining speaker quality somewhat by providing the most generally preferred frequency response for loudspeakers (Olive, 2004). The Harman target has been relatively consistent in representing the preference of a majority of loudspeaker users (Olive, 2004; Olive et al., 2013), and we accordingly used this reference point in Section 3.1 to assess quality and success in our own frequency response tests. Both industry and colloquial spaces perpetuate the Harman target use. An example of this most preferred speaker response appears in Figure 1.

### 2.2 Components of a speaker

**FIGURE 2:**
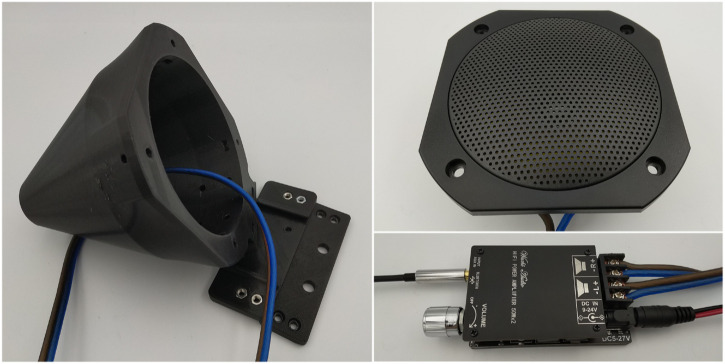
*Left:* Example of a speaker enclosure and mounting plate, disassembled. *Top Right:* Example of a speaker driver (FRS 10 WP speaker driver) with a grille attached. *Bottom Right:* Example of an amplifier module.

Before delving into the specifics of exemplar robot speakers, it is important to set a baseline idea of what speaker design generally entails. Three major components make up a speaker: the enclosure, the speaker driver, and the cross-over network, as shown in Figure 2. The enclosure is the shell that houses the electrical components of the system and is often considered in managing the low frequency responses of the drivers. The speaker driver converts input electrical signals into sound waves. The cross-over network exists to manage multi-driver loudspeakers and separates the high and low frequencies of an electrical signal for reproduction by separate drivers. Other optional components are often present in tandem with the previously listed components. For example, amplifiers increase the overall volume of the input signal and lead to generally “clearer” sound, and ground loop isolators reduce unwanted noise or “buzz” present in the speaker response.

## 3 Broad robot speaker performance and design

In the current robot speaker realm, some robots have built-in speakers, some setups include custom added speakers, and some systems incorporate commercial speakers (e.g., commonly available Bluetooth speakers). As part of this research, we were curious about how well each of these types of speaker setups matched the ideal Harman target, as well as how different options (including both commercial and custom speaker alternatives) compared to one another. Accordingly, Section 3.1 presents an experiment investigating current metrics of robot speakers against a USB speaker alternative. The subsequent experiment in Section 3.2 presents direction for roboticists looking to either improve upon their existing speaker designs or create speakers relative to rigid design constraints. Lastly, Section 3.3 gives guidance on purchasing commercial speakers for robot use cases, for those who prefer to use off-the-shelf options. The associated experiment compares the response of a custom speaker design against similarly-priced USB speakers.

The full range of possible speakers under this analysis umbrella is vast. Accordingly, to make the research tractable, we selected just a subset of the full possible set of speakers in this work, based on hardware and robotic platforms that were available to us. Commercial industrial robots often do not include speakers, so this section tended toward considering speakers for service robots as a feasible comparison point. Similarly, depending on the point in time of the work (for example, during the depth of the COVID-19 pandemic), we did not always have available the same recording equipment or campus spaces. The processes and analysis covered in the present work can be extended to additional hardware and applications; here, we seek to establish a more solid foundation for understanding robot speakers.

### 3.1 Direct comparison of current robot speakers vs. low-cost commercial speakers

To gain a better quantitative understanding of speakers used on current robot platforms, we conducted a brief experiment that compared the use of two built-in speakers from current research robots vs. a commercially available speaker purchased on Amazon at a relatively low cost (18 USD).

#### 3.1.1 Methods

The experiment compared three different speaker alternatives:

• USB speaker (LIELONGREN 8 W) for benchmarking

• Built-in speakers of a Stretch RE2 (Hello Robot, 2024)

• Built-in speakers of a Quori robot (Quori, 2024)

**FIGURE 3:**
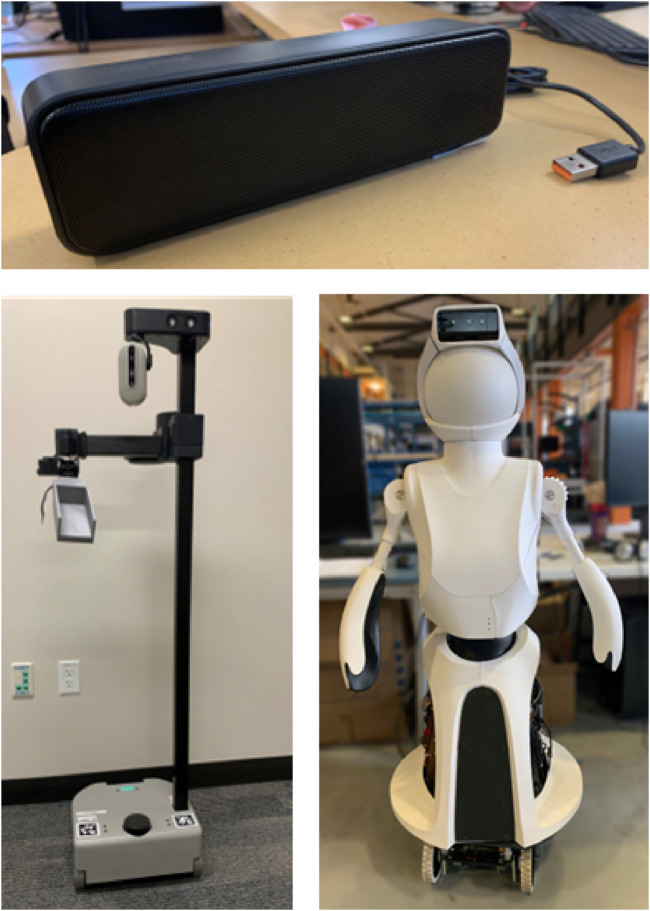
Robots and speaker tested in initial setup. *Top:* LIELONGREN 8 W USB speaker. *Bottom Left*: Stretch RE2. *Bottom Right:* Quori.

We chose the Stretch RE2 and Quori robots for the analysis based on their design for HRI. The Stretch is a commercial robot with a mobile base and arm. The arm has two prismatic joints that help it accomplish manipulation tasks. The Quori robot is a human-sized research social robot with a wheeled base, actuated arms and hip, and a backprojected face. As robots intended to interact with humans, they are likely to use sound as a communication tool. The tested platforms are shown in Figure 3.

**FIGURE 4:**
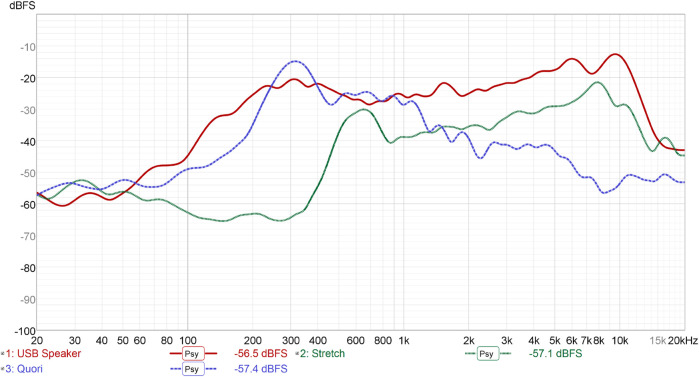
Plot of psychoacoustically-smoothed frequency response curves for the USB speaker, Stretch onboard speaker, and Quori onboard speaker. Initial dbFS values for each curve are represented below the graph.

We used a Dayton UMM-6 USB measurement microphone with Room EQ Wizard to create the frequency response curves shown in Figure 4. In the experiment setup, we placed the speaker drivers a distance of 1 m from the front of the speaker enclosures. The recording environment was a research lab space with a relatively consistent level of environmental noise. For each frequency response curve, we completed a final processing step of applying psychoacoutsics, which removes smaller unheard perturbations in the signal.

#### 3.1.2 Results

The resulting frequency response curves reveal key differences between the three speakers. The USB speaker had a more consistent, flatter ( ±5 dB), and on average louder response when compared to Stretch and Quori, overall appearing to have the best response (i.e., the closest in shape to the Harman target). Beyond the frequency level of 500 Hz, the Stretch speaker also had a relatively flat response ( ±5 dB for 500 Hz–4 kHz). Quori had a relative peak at 300 Hz, followed by a gradual decay afterwards. Qualitatively, when playing pure tones at increasing pitches for the present analysis, we noticed that the onboard speakers exhibited notable crackling and scratchiness that was not present in the USB speaker. These quantitative and qualitative differences would be particularly relevant for speech, whose fundamental frequencies often fall in the 100Hz-1 kHz band. The results of this experiment show a range of quality between the three speakers, including the somewhat surprising insight that a relatively low-cost USB speaker may perform similarly to or better than onboard speakers on modern robots. This result led us to wonder about the potential of custom-made speakers and additionally commercially-available speakers, as further discussed in the following subsections.

### 3.2 Comparison of custom speaker enclosure features

Based on the first experiment’s results, we knew that the speakers of current robotic systems can stand to improve, and we noticed that even commercial speakers leave room for improvement toward the Harman target. Accordingly, we became interested in designing custom speakers. Further, the experiment in Section 3.1 involved only head-on measurements, but real interactions with day-to-day robots can involve additional angles of audio encounter (e.g., standing side-by-side with a robot to share a view of something). These ideas, taken together, led us to experiment with custom speakers that varied enclosure shape, as a potentially promising way to modulate range of audibility.

At a basic level, a speaker enclosure is the housing around the speaker hardware component itself, which impacts how the speaker’s sound interacts with the surrounding environment. We developed a variety of 3D-printed speaker enclosure designs to consider potential custom-built enclosure performance for different use cases. For simplicity, we used a sealed enclosure design for all speaker prototypes. We varied shape in our base design, seeking to directly compare the output of different shapes of enclosure. We also collected baseline data from a no-enclosure design. For the square design, one considered shape which matches speakers most commonly found in robotic systems, we modulated the shape of internal corners, presence/absence of infill, and presence of grilles.

#### 3.2.1 Methods

**FIGURE 5:**
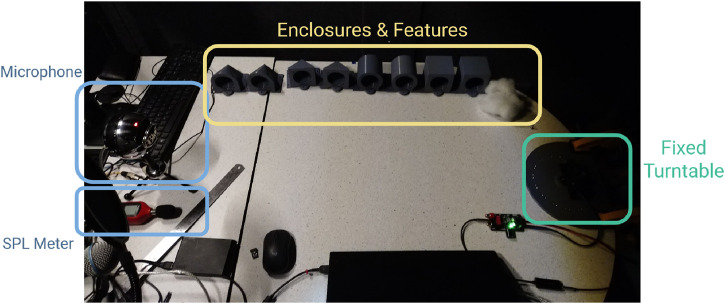
Speaker testing setup, including the range of tested speaker enclosures and a turntable for fine adjustments to speaker direction.

We performed this experiment in a semi-anechoic (i.e., noise-absorbent other than the floor and ceiling) room. For each enclosure design, we recorded the output from the same speaker driver (i.e., a MISCO Oaktron 93,003 powered by a DROK 5 W amplifier breakout board) playing 20 kHz–20 Hz sweeps generated by the program Audacity. We rotated the speakers incrementally between 0° and 180° by 22.5° to test for directivity. A Blue Snowball USB microphone, set up on the same table 1 m away from the speaker, recorded the resulting sound. Figure 5 shows the test setup.

The enclosure shape alternatives tested in this experiment were:

• no enclosure (i.e., bare)

• a square enclosure

• a cylindrical enclosure

Within the common square enclosure type specifically, we considered:

• a square enclosure with no extra features

• a square enclosure with filleted internal corners

• a square enclosure filled with Poly-Fil (a common type of speaker infill)

• a square enclosure with a protective grille (i.e., capped)

The shapes of the enclosures were chosen based on a combination of early pilot work that explored a wider range of shapes and insights from commercial speakers that often employ these specific form factors. We 3D-printed the enclosures with PLA on a Prusa MK3S. All enclosures had a characteristic internal dimension of 67 mm (i.e., 67 mm deep, with a 67 mm diameter or width) and 4 mm-thick walls. Our data analysis used MATLAB’s FFT function to produce the resulting frequency response curves. The Fourier transforms were converted to dbFS byapplying Equation 1 to its output:

$$20\times \log_{10}\left(|\upsilon_{\mathrm{freq}}|/ref\right)$$ (1)

Here, $\upsilon_{\mathrm{freq}}$ represents the amplitude at a particular frequency and ref was the maximum value of the Fourier transform. The resulting graphs were smoothed using MATLAB’s smooth function.

To avoid computer-specific interference, audio was outputted from a Creative Labs Sound Blaster Play 3 USB DAC. Further, to avoid potential hiss due to ground loop problems, audio was passed through a BESIGN ground loop isolator.

#### 3.2.2 Results

**FIGURE 6:**
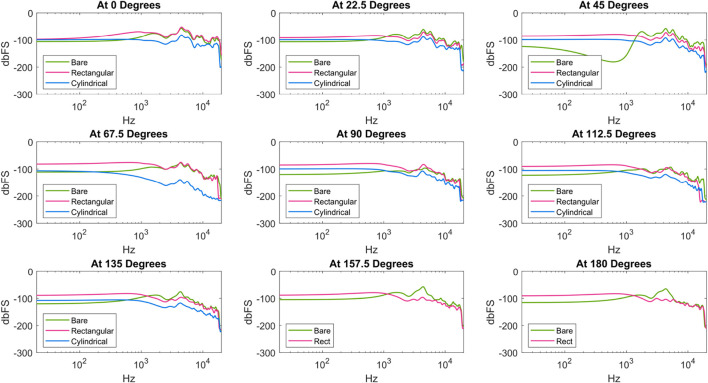
Frequency response of speaker enclosures across shape. All data is included except for two cylindrical recordings, during which we experienced a data recording error.

**FIGURE 7:**
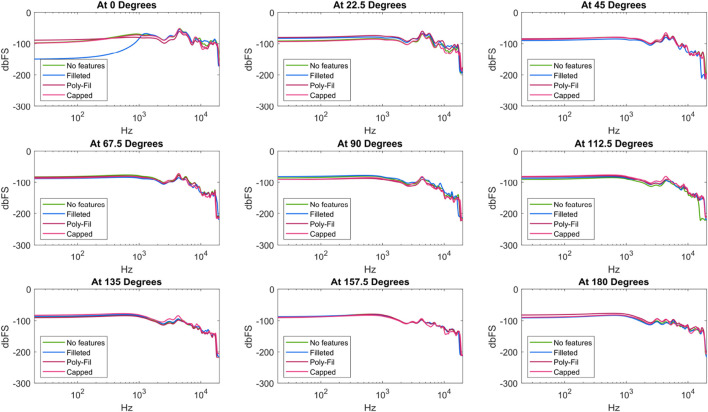
Frequency response of speaker enclosures across filling and feature type.

Figures 6, 7 show the results of the speaker shape and feature testing, respectively. At a high level, the clearest observed differences between enclosure options were due to shape. The rectangular shape performed the best out of the different shapes considered, as evidenced by the generally higher values it sustained at lower frequencies. While there were frequencies for which the bare condition outputted a higher volume, the rectangular condition had a much more consistent and relatively flatter profile throughout the tests (i.e., more similar in overall shape to the Harman target by comparison). The bare and cylindrical speakers notably exhibited spikes and troughs during disalignment, as seen at 45° and 67.5° degrees, respectively. For other speaker features (e.g., fillings, grilles), there were few distinctions between the considered enclosures. The most notable difference was for the filleted enclosure, for which the loudness of the non-rotated speaker was negligible until around 1 kHz.

The results showed a potential benefit of larger enclosure sizes. The square enclosure, which afforded the largest internal volume within the same square footprint, had the most consistent frequency responses regardless of direction, with higher volumes overall. If roboticists are seeking to create custom speakers, maximizing or attempting to use as much space as possible for the speaker enclosure should be a priority. Considering the limited effect that internal fillets, Poly-Fil, and grilles had in the direct tests, roboticists can prioritize other elements of design over these features.

### 3.3 Comparison of commercial and custom speakers

We were pleasantly surprised by the quality of performance of the low-cost commercial speaker in Section 3.1, and we considered that this type of off-the-shelf speaker may be a good option for researchers seeking to add sound capabilities to robots. At the same time, we wondered if our choice of speaker was a fortunate pick, or if a range of speakers would offer a similarly good performance. We also were curious if a speaker similar to the best-performing custom speaker from Section 3.2 would offer advantages (or disadvantages) compared to commercial speakers.

Accordingly, our next step was to compare the performance of a broader range of off-the-shelf speakers against a custom speaker with a slightly increased internal dimension compared to the top performer in Section 3.2 (ideally to accomplish an even better performance). Off-the-shelf speakers offer the potential advantage of not requiring as much setup and individual component purchase compared to custom speakers. On the other hand, if accompanied by a corresponding improvement in sound production, custom speakers could still offer value, especially in cases when off-the-shelf parts do not fit into a robot’s construction (as one example).

#### 3.3.1 Methods

For this experiment, the frequency responses of the following five speakers were captured using a Dayton UMM-6 measurement microphone and Room EQ Wizard (REW):

• Creative Pebble speakers, connected via 3.5 mm cable.

• LIELONGREN 8 W sound bar speaker, connected via USB.

• LIELONGREN cube speaker, connected via USB.

• HONKYOB mini stereo speaker, connected via USB.

• A custom speaker with a characteristic internal dimension of 80 mm and 4 mm-thick walls

We chose the listed commercial speakers based on their price range. On retail sellers, these options were priced at 8–20 USD. The electrical setup for the custom speaker was identical to that used in the experiments in Section 3.2. The testing setup was similar to in the past subsection, with each speaker placed on-axis a distance of 1 m from the recording mic, on the surface of a desk in an office environment. A Class 2 sound meter with a measurement range limited to 10 kHz (to avoid speaker blowout) was used to tune the volume of the speaker. All software effects for both the speakers and microphone were turned off in Windows.

#### 3.3.2 Results

**FIGURE 8:**
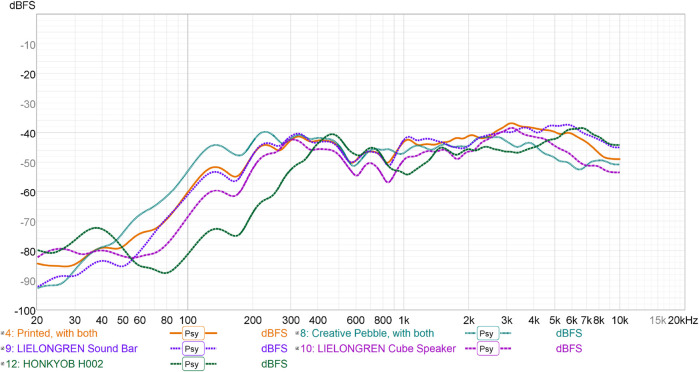
Psychoacoustically-smoothed frequency response curves for the studied USB speakers and custom speaker.

Figure 8 shows the resulting frequency response curves. This plot shows that the Creative Pebble speakers performed best at low frequencies, while the custom speaker, sound bar speaker, and mini stereo speaker performed best at high frequencies. The largest differences between the responses and the Harman target occurred at low frequencies, but relatively asymptotic behavior was consistently achieved by all speakers after 300 Hz except the HONKYOB H002. Overall, consumer speakers sized like to the custom speaker performed similarly well, while smaller speakers (i.e., the cube speaker and mini stereo speaker) performed worse in the lower frequencies. Qualitatively, the Creative Pebble speaker also occasionally cut out, particularly at louder volumes and higher frequencies. The USB speakers did not have this problem, likely due to their ability to digitally limit the acceptable volume to remain within appropriate amplifier and speaker driver ranges.

### 3.4 Summary of key results

One main takeaway from this section is in the recognition of the possible faults that exist in current robot speakers. We present and evaluate custom speaker options, focusing on the speaker enclosure to supply new options for remedying existing problems in the frequency response of current robotic platforms’ speakers. For custom speakers, we found that internal volume should be maximized in order to yield flatter frequency responses, and filling does not seem to be of great importance (despite it being common in speaker designs). Alternative and low-cost USB speakers could be viable alternatives with comparable performance to the custom speakers, especially if they are of a similar size to the custom design.

For a roboticist seeking the simplest and most reliable solution, a larger USB speaker may satisfy their needs. For a roboticist needing to satisfy specific design requirements or applications, custom speakers with appropriate design considerations may be needed. We explored two such cases in Section 4.

## 4 Custom speaker design case studies

Depending on the robot application, it can be important to incorporate custom speakers to achieve certain design criteria (e.g., audible warnings in safety-intensive scenarios, pleasant-sounding voice frequencies in social interactions). Often, these speaker additions are needed in settings that may not be compatible with commercial speaker solutions (e.g., harsh environmental conditions, interactions with sleekly designed social robots). To better understand these scenarios and to give further examples of how custom speakers can be implemented with different robotic hardware, we present two separate speaker design case studies. The first gives a practical example of how custom speakers can be tailored for a robot in an industrial construction setting (Section 4.1). The second (Section 4.2) presents qualitative feedback on a custom speaker kit that we developed for augmenting current social robots.

### 4.1 Design 1: Construction robot speakers

Robots operating in industrial construction sites have unique requirements if they want to be able to use sound as a mode of communication. An existing study from our own past work sought to understand the sound equipment and profile requirements necessary for robots in construction settings (Frazee et al., 2020) (presented as a workshop paper). This work centered around the use of a Husky robot, a rugged commercial mobile robotic system often used in the construction industry for logistical monitoring (Bogdon, 2019a; Bogdon, 2019b). We wanted to equip this robot with appropriate sound-based communication for its usual operating settings.

Domain-relevant specifications informed the selection of individual components in the speaker driver, design of the speaker enclosure, and speaker amplifier in this past work, as further detailed below. The design of the speaker only had a single driver, so a cross-over network was not considered.

#### 4.1.1 Sound equipment requirements

Based on standards and related work in the construction domain, we identified the following requirements for our custom speakers. Our speakers needed to:

1. Withstand common environmental conditions. The equipment should have Ingress Protection (IP) ratings ranging from IP 55 to IP 68 to reflect the range from the Husky robot (Clearpath Robotics, 2020) to buzzers and alarms designed for construction sites (Brigade Electronics, 2020).
2. Produce sound at an audible level. The equipment should sustain a maximum sound pressure level (SPL) of at least 112 dB for tonal output, or at least 107 dB for broadband. This requirement was determined based on specifications for worksite buzzers and alarms (Holzman, 2011; Brigade Electronics, 2020).
3. Interface with existing components. The Husky robot offers power interfaces at 5 V, 12 V, and 24 V with 5 A fuses. Additionally, the onboard computer offers common computer peripherals, such as USB ports and a 3.5 mm audio jack (Clearpath Robotics, 2020). The audio system should interface with the robot’s power and inputs directly. The authors chose a speaker system over other options such as buzzers and surface transducers due to limited sound frequencies for both options and limited surface for vibration in the latter.

#### 4.1.2 Speaker hardware

Speaker Driver: We focused on the production of broadband sound based on its effectiveness in past work (Cha et al., 2018). Accordingly, we sought a driver capable of a sustained SPL of approximately 107 dB at 1 m. Based on the aforementioned specifications, the FRS 10 WP (Visaton, 2015) was chosen. Based on the speaker driver’s specifications of 25 W rated power and 90 dB mean SPL at 1 W and 1 m, the speaker driver can provide up to 104 dB according to Equation 2 (the sound power level equation), in which L is the SPL, P is the sound power (units of pW) produced, and $P_{0}=1\text{ pW}$ is the reference sound power in air (Self et al., 2009).

$$L=10\log_{10}\left(\frac{P}{P_{0}}\right)\text{dB}$$ (2)

We anticipated that a two-speaker system, which would also allow for more complex signaling through stereo sound, would provide the required 107 dB. Due to the inverse-square law, there is potential for the proposed audio system to produce hazardous levels of sound (i.e., 120 dB or above) if the listener is less than 0.16 m away. It is recommended that a safety barrier or cautionary signage be included with the proposed audio system.

Speaker Enclosure: The speaker enclosure was designed based on recommendations outlined in Section 3.2. We sought to maximize internal volume without an excessive footprint, selecting a footprint of 125 mm long and 115 mm wide instead of the exceedingly large manufacturer-recommended enclosure volume of 2 L (Visaton, 2015). The enclosure was 3D-printed using polylactic acid (PLA). In order to improve the acoustics of the speaker, the enclosure was filled with a polyester filament based on recommended practices from past work (Dickason, 2006). The enclosure coupled with a custom mounting plate (as shown in the left side of Figure 2) that clamped to the top mounting plate of the Husky. This design can easily be adapted to mount to other robots.

Speaker Amplifier: Our system requires an amplifier to convert low-power audio signals from the Husky computer to high-power audio signals for the speaker drivers (Self et al., 2009). We searched for a stereo amplifier breakout board with power specifications similar to the speaker drivers but below the maximum power output of the Husky robot to avoid tripping the 5 A fuse. This led to a maximum total power rating of 120 W from the 24 V power supply. Thus, we selected the TPA3116D2 amplifier breakout board, a component capable of driving two 50 W outputs (Texas Instruments, 2020).

#### 4.1.3 Evaluation

**FIGURE 9:**
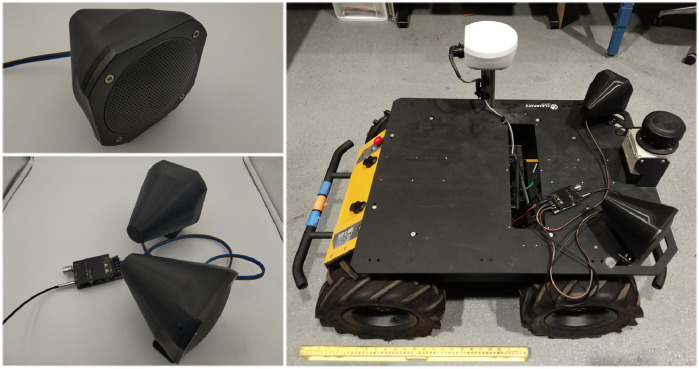
*Top Left:* the fully assembled speaker enclosure. *Bottom Left:* two-speaker system with the FRS 10 WP speaker driver and the TPA3116D2 amplifier module pictured fully connected. *Right:* Husky Robot with full system assembled.

**FIGURE 10:**
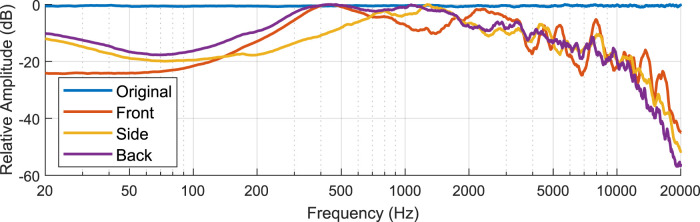
The recorded frequency responses compared with the originally played broadband noise. The data was analyzed with the Plot Spectrum tool in Audacity with size 65,536 and smoothed with a moving average filter with window size 100.

The prototype system was installed on a Husky robot, as illustrated in Figure 9. Using a Blue Snowball microphone, we recorded the system playing white noise at maximum power from three locations: 1 m from the front, left side, and back edges of the robot. The loudness, power requirement, and frequency responses at the three positions were extracted from the recording. The system produced white noise with a mean SPL of 107.8 dB in front of, 101.0 dB to the side of, and 97.7 dB behind the robot. Based on Equation 2, the system drew at least 59 W from the robot. Figure 10 shows the frequency response of the recording in each position compared to the original broadband noise waveform being played through the speakers.

Relevant frequency ranges needed for intentional robot sound in construction appeared to be strong in our results. In particular, frequency responses in the ranges of 400–800 Hz and 1,600–3,500 Hz, which have proved promising for localization in past work (Holzman, 2011), are strong The results also support the idea of using broadband sounds for warning; tonal sounds could be less effective due to the peaks and troughs present in the frequency response results, which would more strongly impact their audibility (compared to broadband signals).

Overall, the system satisfied the specifications successfully, with the required SPL of 107 dB at 1 m away in the forward direction being achieved. Notably, however, factors such as acoustic absorption, speaker directivity, and speaker placement appeared to negatively affect the resulting SPL on the side of and behind the speakers, resulting in an SPL below the target in these other locations. It should be considered whether additional speakers or alternative orientations could be useful depending on application and typical positioning of people relative to the robot on a daily basis.

The speaker power consumption of 59 W may also prove to be an issue due to potentially reducing the runtime of the 160 W Husky robot. This power consumption could result in a runtime reduction of up to 27% and could be detrimental to the fuses of the 5 V and 12 V supplies as well. Thus, the power supply capabilities and points of access should be carefully considered before installing the proposed speaker system. However, this reduction is a worst-case scenario that assumes that the speakers are running at full power at all times.

### 4.2 Design 2: Social robot speakers

Using consumer robots as research platforms has grown increasingly popular as robots become commercialized and gain broader use. While consumer robots can lead to faster and potentially more interesting research, they often limit direct control of the robot’s motors (and other hardware elements, including speakers), as most consumers do not require this type of low-level access. These barriers can be a concern for roboticists seeking to investigate nonverbal sounds not included in the robot’s API or not easily accommodated by it. As one example of this type of access challenge, another research team at the KTH Royal Institute of Technology required external speakers to be able to study sonification techniques using Pepper, a sleek commercial robot that is human-sized and has a chest screen for interaction and actuated limbs, head, and waist. We developed and shared a custom speaker with them as part of a 2022 IEEE International Conference on Robotics and Automation (ICRA) workshop effort, and we worked with them to assess speaker outcomes, as further detailed below. Our goal in this work was to facilitate the introduction of easy-to-use and audible modular speakers for need scenarios like that of the KTH team. This effort was approved by the Oregon State University Institutional Review Board under protocol #IRB-2021-1154.

#### 4.2.1 Sound equipment requirements

Based on conversations with the KTH researchers, we established this set of design criteria for the custom Pepper speaker hardware. The hardware needed to:

1. Be modular. The design of the system should be fairly compact and modular to allow for a variety of possible configurations on Pepper or other similar social robots (for good potential reusability/broader adoption).
2. Interface with existing components. In order to be integrated relatively quickly on existing commercial robot platforms, the system needs to easily interface with common I/O methods.
3. Be audible in common social robot settings. Social robots often use speech, whose fundamental frequency can range from 100 Hz to 1 kHz. This range is needed to be audible in common use settings.

#### 4.2.2 Speaker hardware

**FIGURE 11:**
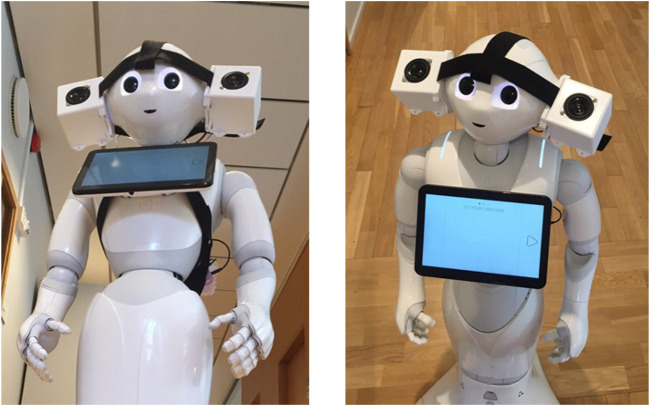
The Pepper robot with the custom speaker system strapped to its head.

We used our previous work on speaker enclosures to develop a speaker system that could be easily attached to or removed from a robot without built-in mounting points. The original enclosure design was modified to include a lower plate that could be attached or detached; together, the enclosure and lower plate clamped onto a strip of Velcro. This Velcro-based attachment system enabled the end user to select and adapt the placement of the speaker system as desired. Figure 11 shows the assembled speaker system on the Pepper robot.

To facilitate the incorporation of the system with the KTH Pepper robot, we created a part kit including the unassembled speaker components and an instruction manual. After our collaborators received our package, they assembled the speaker system without our assistance and conducted sound-based experiments with the system. As a closing element of this work, we conducted a semi-structured interview with our collaborators to identify key strengths and limitations of the speaker system.

#### 4.2.3 Evaluation

The feedback from the semi-structured interview was promising for future iterations on the modular speaker kit concept. The collaborators noted that the kit “was really easy to assemble” due to only requiring two tools for the full assembly. The users noted that the speakers provided reasonable sound quality. Based on video demonstrations of the system, the Pepper head appeared to maintain its full range of motion capabilities with the speakers installed. Based on this assessment, we believed that we generally accomplished our high-level goals.

We also asked the collaborators about how the kit could be improved. One point of critique was that although the current enclosure was 3D-printed out of plastic, there is potential to instead use wood like many traditional loudspeakers. Although properties of the enclosure influence the tone produced by the speakers, the collaborators “did not [feel] that [they were] missing so many important frequencies.” The users had interest in future implementations that could produce directional sound output.

### 4.3 Summary of key results

Custom speakers were shown to have viability in two design case studies with a construction and a social robot. Overall, custom speaker systems provide robust flexibility for adapting to specific use cases. In the industrial construction domain, the custom speaker allowed for a tailored approach to meet specific robustness, sound level, power, and I/O requirements needed for the setting. For the social robot, the custom speaker kit enabled more flexibility than the robot’s existing construction and API. Overall, custom speakers show promise for filling in gaps when commercial speakers fail to satisfy sound-related design needs.

## 5 Human subjects study on speaker quality for voice

As a more formal extension of understanding sound design requirements for service robots, we conducted an experiment that sought to explore what effects levels of speaker quality have on views of a robot’s social attributes, localizability, and value. This section focuses on service robots due to them commonly possessing onboard speakers, but we believe the results can be relevant to industrial robot design in interactions such as collaborative tasks. All study procedures were approved by the Oregon State University Institutional Review Board under protocol #IRB-2020-0592.

### 5.1 Study design

During this within-subjects study, the participant worked with a Hello Robot Stretch RE2 robot, which is intended for personal and service applications.

#### 5.1.1 Space setup

**FIGURE 12:**
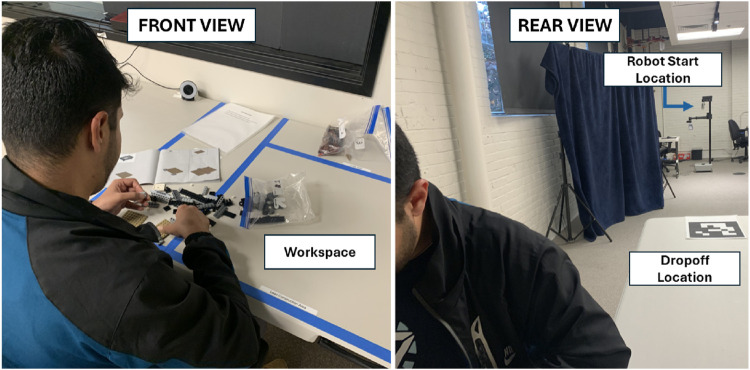
The study setup. The left image displays the participant workspace and perspective, and the right image shows the robot starting location, a curtain that obscures the view of the researcher, and package dropoff location.

Figure 12 shows the study room setup. A webcam was located in the human’s workspace, along with starting materials for the study task.

The participant engaged in a LEGO assembly task while facing away from the robot, and the robot intermittently visited the workstation to drop off bags of LEGOs, one in each trial, to support the continued assembly. When dropping off a package during sound-added conditions, the robot played the following statement upon arrival at the participant’s desk: “I have a package here for you.” After completing its task, the robot would departed and (in the sound-added conditions) played the statement: “Good job, keep it up.” Voicelines were generated using Amazon Polly in March 2023 using default settings: neural engine, US English, and the Joanna voice.

#### 5.1.2 Central manipulation

The study conditions involved three different types of sound scenarios:

• No added sound: a baseline condition. No statements were played.

• Onboard speaker sound: the Stretch RE2’s built-in, custom speakers, located at the top of the robot, played the statements.

• Added speaker sound: a commercial LIELONGREN 8 W USB speaker affixed to the top of the robot played the statements.

The robot had no visual differences across the three trials (the added speaker was always affixed, for control).

### 5.2 Participants

21 participants were recruited through Oregon State University email listervs and successfully completed the study. Participants were aged 18–61 years ( M=32.5 , SD=16.0 ), with 47.6% men (including 4.8% transgender men), 47.6% women, and 4.8% nonbinary individuals. Four participants declined to share their age. Previous robot experience was reported as follows: 42.8% claimed to have general awareness of similar products, 14% had researched or investigated robots, 4.76% have participated in a demo of a robot, and 28.5% were not aware of a similar product.

### 5.3 Procedure

Upon entering the study room, the participant was asked to provide informed consent. Next, the participant was instructed to build a LEGO set while seated at a dedicated workspace and move any bags delivered by the robot in the study space to their workspace. They started with with the first three bags of the set already in their workspace. Next, the study condition experiences, presented within-subjects in a counterbalanced order, began. About 2 min after the participant started their task, the robot approached to deliver additional LEGOs, playing or not playing designated drop-off and departure statement depending on the condition. A research assistant brought the participant to a different table to complete a post-trial survey after each delivery. This overall process repeated for each condition. Following the final trial, participants completed a demographic survey and semi-structured interview.

### 5.4 Measurement

Our post-trial survey collected self-reported information about robot social attributes, localizability, purchasing interest, and value. We used the Robotic Social Attributes Scale (RoSAS) (Carpinella et al., 2017) to measure perceived warmth, competence, and discomfort during interactions with the robot on a 6-pt Likert scale from “Definitely Not Associated” to “Definitely Associated” (omitting a neutral scale option to force decisions). Participants reported robot localizability using level of agreement with a custom item (i.e., “I could tell where the robot was at all times.”) on a 6-pt Likert scale from “Strongly Disagree” to “Strongly Agree.” We captured information about value using the Price Sensitivity Meter (PSM) (Breidert et al., 2006). This inventory assesses user purchasing interest on a 5-pt Likert scale from “Not at all interested” to “Extremely interested,” in addition to four dollar-valued price points: too cheap, cheap, expensive, and too expensive.

We conducted a semi-structured interview with participants at the end of the study. Participants answered questions about their impression of the robot and what aspects of the robot influenced their survey responses. The end-of-study interview also included questions about participants’ favorite condition (from memory), as well as an opportunity to expound on differences between conditions. Next, the speakerconditions were replayed to the participant, accompanied by questions about their favorite condition, opinions on the differences in voice, and whether participants were able to guess the premise of the study before it was revealed.

A final demographic survey recorded participants’ age, gender, ethnicity, nationality, hometown, profession, robotics experience, and musical experience.

### 5.5 Hypothesis

Generally, we expected a higher-quality speaker to be most favorable. Accordingly, our main hypothesis was that:

Hypothesis 1 Using a higher-quality speaker for speech-based human-robot interaction will lead to greater warmth, competence, purchasing interest, perceived value, and preference when speaking compared to a lower-quality speaker.

### 5.6 Analysis

The post-trial survey questions were evaluated using repeated measures analysis of variance (rANOVA) tests with an α=0.05 significance level. Concerns such as spherecity violations were considered through use of the Greenhouse-Geiser corrections. In the case of significant main effects, we used Tukey’s Honestly Significant Difference (HSD) test to identify significant pairwise differences. In the case of non-normality, Friedman’s tests were conducted with Holm-Bonferri corrections. With the rANOVA tests, the effect size was reported using generalized eta-squared $\left(\eta_{G}^{2}\right)$ to improve cross-study comparability (Olejnik and Algina, 2003). Effect sizes were interpreted relative to Funder and Ozer’s updated guidelines (Funder and Ozer, 2019) after Cohen’s original work (Cohen, 1988). Financial value responses were further assessed through the PSM analysis methods detailed in prior work (Breidert et al., 2006; Zhang et al., 2022). All statistical analyses were conducted using jamovi, a graphical interface for statistics powered by R, as well as open-source modules developed for jamovi (The jamovi project, 2021; R Core Team, 2021; Singmann, 2018; Lenth, 2020).

We performed thematic coding on the qualitative data from the semi-structured interviews. We also tallied responses to the conditional preference questions from the interview.

### 5.7 Results

27 participants enrolled in the study. Due to robot failure and researcher error, six participants’ partial data was excluded from analysis. 21 participants in total completed the full study.

#### 5.7.1 Social perception and localizability results

The results of the rANOVA tests showed significant differences in RoSAS responses for:

• Perceived warmth ( F(1.41, 28.30)=14.70 , p<0.001 , $\eta_{G}^{2}=0.249$ )

• Perceived competence ( F(1.63, 32.58)=3.72 , p=0.043 , $\eta_{G}^{2}=0.042$ )

Pairwise comparisons showed that the onboard speakers had greater warmth than the condition with no added sound. The added speakers were also found to be warmer than the condition with no added sound. There was no singificant difference between the two speaker conditions.

While the rANOVA test results for competence yielded significance, no significant pairwise differences were found (all p>0.072 ) after the Tukey’s HSD test. The differences between the onboard and no-added-sound conditions (p=0.072) and the added-speaker and no-added-sound conditions (p=0.084) were marginally significant. The added-speaker condition tended to be rated the highest, followed by the onboard-speaker condition and then the no-added-sound condition.

**FIGURE 13:**
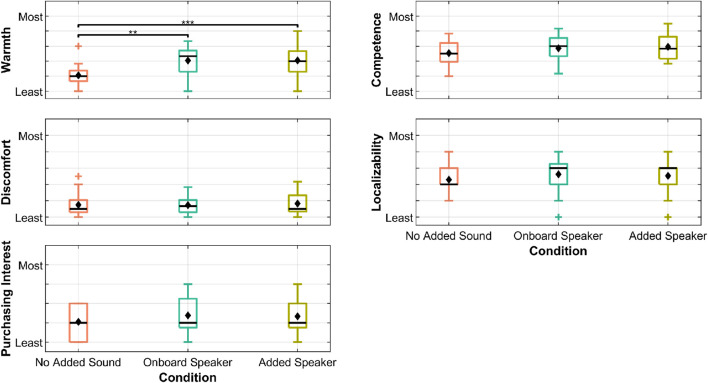
Boxplots of study results for the three scales that yielded a significant main effect. Black horizontal lines represent the median, diamonds represent the mean, pluses represent outliers, boxes represent the range from the 25th to the 75th percentiles, and whiskers cover up to 1.5 times the interquartile range. Brackets represent significant differences. Asterisks above the brackets indicate the level of significance: * for p<0.05 , ** for p<0.01 , and *** for p<0.001 .

There were no significant main effects in discomfort or localizability ratings (all p>0.2 ). An overview of Likert-based results are shown in Figure 13.

#### 5.7.2 Perceived value results

The results of the rANOVA tests indicated a significant difference for perceived value:

• Purchasing interest ( F(1.79,35.81)=3.43 , p=0.048 , $\eta_{G}^{2}=0.023$ )

Despite the significant main effect, no pairwise differences were found to be significant (all p>0.074 ) after the Tukey’s HSD test. The difference between the added-speaker and no-added-sound conditions was marginally significant (p=0.074) . The added-speaker condition tended to have the greatest perceived value, followed by the onboard-speaker condition and then the no-added-sound condition.

Two price tiers were found to yield significant differences after a Friendman’s test with Holm-Bonferroni corrections:

• Expensive ( $\chi^{2}=11.40$ , p=0.003 )

• Too expensive ( $\chi^{2}=7.85$ , p=0.023 )

Pairwise comparisons with Holm-Bonferroni corrections revealed that the expensive pricing was significantly greater with the added speaker compared to both the onboard-speaker and no-added-sound conditions. The other pairwise comparisons did not yield significant differences.

In the too expensive pairwise comparisons, the too expensive value for the added-speaker condition was significantly higher than the value for the no-added-sound condition. The other pairwise comparisons did not yield significant differences.

**FIGURE 14:**
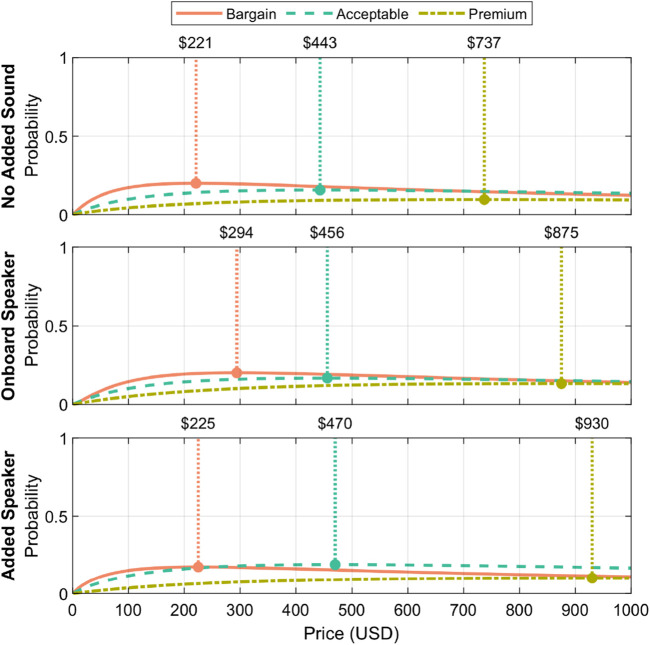
Extended PSM analysis results for the no sound added, onboard speaker, and added speaker conditions. The curves fitted during this analysis highlight recommended price points for bargain, acceptable, and premium pricing, with the premium price point in particular seeming most robust based on our findings.

As a result of the identified significance in the PSM input, we performed an extended PSM analysis on the study data; Figure 14 shows the results. Two participants were excluded from the PSM analysis due to non-monotonically-increasing answers (i.e., ratings of all zeros, or inputting a cheap price that is greater than an expensive price). As the “too cheap” and “cheap” responses did not yield significant differences, care should be taken in considering the “bargain” and “acceptable” price points. We consider the “premium” price point to be robust, based on the significant results discussed in this subsection.

#### 5.7.3 Thematic coding results

Qualitative data from the semi-structured interview was coded and grouped according to the facets and themes illustrated in Table 1. Specifically, the themes in participant responses were: (1) condition differences, (2) personification of the robot, (3) “robotic” characteristics, and (4) implications of no sound. Themes 1 and 2 included two facets each, while the other themes mapped directly to just one facet.

**TABLE 1:** Results of the coding, including theme numbers, facet (or code) names, example quotes, and frequencies. We use the abbreviations “S” for no added sound, “O” for onboard speaker, and “A” for added speaker.

Theme	Facet	Example	Frequency
1	Awareness of Conditions	“the only differences I noticed in the robot were […] or more like, whether it played a message or not”	11 (all S)
1	Comparisons Against Silent	“when it added in the voice and encouragement […] added the personal attributes to it”	8 (all S)
2	Personification of Speech Quality	“I thought [added speaker] sounded friendlier […] more genuine and more realistic like I was talking to a person”	13 (12 A, 1 O)
2	Personification of Attributes	“the robot talking made it seem more friendly, made it seem more human-like and compassionate”	2 (all S)
3	Expectations of Robot Speech	“I thought the [onboard speaker] sounded more automatic and more robot-ish”	10 (all O)
4	Implication of Consequential Sounds	“[consequential] it’s even alarming to me, […] does not tell me what it’s up to”	7 (all C)

Under Theme 1, n=11 participants commented on the apparent differences (or lack thereof) between conditions. Two participants among this group noted the exact right distinction between the trials. For the second Theme 1 code, n=8 participants commented on the interactivity of the robot as a key element of distinguishing between trial experiences. Under Theme 2, the personification of the speech quality of the robot included comments made after the reveal of the conditions (n=13) . These notes were generally focused on the added speaker ( n=12 of the set). The personification of robot attributes generally ( n=2 comments) was more broad and took into account personifying comments about the robot’s speech in general. The noting of “robotic” speech or what qualified as such was common when participants were comparing the onboard speaker and added speaker (n=10) . Overall, there were mixed comments about the lack of speech for the no-added-sound condition, mostly surrounding either descriptions of its practicality or other negative connotations like how alarming it was (n=7) .

#### 5.7.4 Condition preference results

When asked about their condition preference, 71.4% participants reported the added-speaker condition as their preference, 23.8% reported the onboard-speaker condition, and 4.8% reported the condition with no sound added.

### 5.8 Summary of key results

Based on the results, Hypothesis 1 was partially supported by the pricing results and the price points resultant from the PSM analysis; the acceptable and premium pricing for the added-speaker condition were compared to the same price points for any other condition. Additionally, there was good evidence of a benefit of speaker presence in general. For example, warmth and competence ratings were highest for any robot with a speaker. This finding is further supported by the results of the thematic coding, where robot speech in general seemed to be a driving factor of positive ratings of the robot. It is not clear how much the differences between the two speaker types influenced participants; participants were typically not aware of the differences between the two speakers before the subject was briefed in the interviews. Only 2 participants identified that the speaker characteristics changed during the experiment, i.e., before the conditions were revealed and replayed in the second half of the interview. Once the speaker condition were revealed and laid out, giving participants an opportunity to engage with the robot sounds without the distractor task of LEGO assembly, there was an emergent predominant preference for the added speaker. Most participants preferred this added speaker during the interview discussions, with justifications such as the emerging voice sounding more “human,” “human-like,” or “smooth” than the more “robotic” or “scratchy” onboard speakers.

## 6 Discussion

Over the course of this work, we improved knowledge about the current quality of robot speakers, introduced design ideas for custom speakers made to fit specific needs, investigated two cases of custom speaker use in common robot application scenarios, and conducted an in-lab study on the effects of different types of robot speakers on human-robot interaction.

### 6.1 Recap and discussion of broad robot speaker performance and design

A key notion from the results of Section 3.1 is that the quality of current robotic speakers is not on par with that of even low-cost commercial speakers. The results of Section 3.2 showed that speaker enclosure shape can have a visible impact on sound quality, although other common speaker features did not make as big of a difference on frequency response readings. A key insight from the effort generally is that more enclosure volume tended to do better. Section 3.3 showed promise in our custom speakers compared to USB speakers at similar price points. At the same time, custom speakers themselves are not always ideal considering the relative knowledge and engineering requirements associated with their deployment.

*Limitations* of this part of the effort include the fact that this segment of the work borders more on technical evaluation of hardware than research. Yet, the topic of speakers is unfamiliar to many roboticists, and we sought to provide clearer definitions, sound profile targets, and general rules of practice to those who may not yet have experience with speaker-related topics. We are fortunate to have researchers from both the robotics and sound disciplines working on our team, and we want to spread the advantages gained from this interchange to other robotics researchers. Our analyses of the frequency responses of the loudspeakers were fairly simple in nature. Due to lacking access to an aneholic chamber, we made compromises such as studying speakers in a semi-aneholic room. However, considering the target audience who will usually have similar access restrictions, the accessible and reproducible methods presented may be optimally useful for building a foundation for future related efforts by roboticists.

### 6.2 Recap and discussion of custom speaker design case studies

To inform processes in cases where custom speakers are justified, Section 4 highlights example methods and case study results for two custom speaker design challenges. Section 4.1 considered the case of requirements based on the challenging environment of a construction site, where speaker hardware needs to survive harsh conditions (unsuitable for much commercial sound hardware) in addition to being audible. We presented a custom speaker solution that was suitable for the industrial construction environment in addition to fitting the identified sound level requirements when experienced head-on. In the social robotics domain discussed in Section 4.2, where “plug and play” use of sound hardware can be infeasible due to hardware constraints of robots, we demonstrated an adaptable modular speaker kit that met our collaborator’s needs in the studied case. We encourage readers to similarly evaluate the potential of custom speaker hardware in further cases where interfacing with existing commercial hardware may be impossible or impractical.

*Limitations* of this work include a limited number of cases and examples considered. We know that the design needs and impacts for different robotic platforms, interaction contexts, and user settings will come with their own unique requirements.

At the same time, we believe that the presented work can serve as an informative example of how to elicit design requirements, develop hardware, and evaluate hardware in a way that can support forward progress in robotic sound, and especially within the realm of speakers for robots. We reiterate that buying commercially available speakers will likely be the smoothest route for adding speakers to many robots; the need for custom speakers will depend on robot and context. Custom speakers should most likely be considered in cases of strict hardware constraints (e.g., power draw) or limited I/O available for USB- or jack-based commercial options.

### 6.3 Recap and discussion of human subjects study on speaker quality for voice

The results of Section 5 provided direct insights on implications of robot speakers in service robotics domains (as one example case in which speakers are already relatively common). The findings of this empirical study suggested that the quality of a speaker had less effect on the interaction than the presence of added sound generally (counter to the expectations in this study’s hypothesis). We did see some effects of the added-on speaker compared to the onboard speaker, specifically in evaluations having to do with monetary value. Our recommendation based on this study is that robot ability to use speakers may be more important than the specific speaker used, up to a point. The results overall hint at social, functional, and value benefits of robot speakers.

*Limitations* of this part of the work include a relatively small sample size, as well as some of the same ideas as mentioned in the last subsection. Similar work is needed for robots of more types and in more use cases to fully understand the area of robot sound. Future studies could also consider comparisons of more speaker quality conditions within the same experiment design, in addition to expanding the use of the types of sounds (beyond voice) explored. Additionally, study of longer-term interactions would likely yield even more ecologically valid conclusions. For example, occasionally a sound that is acceptable at first becomes annoying or unpleasant to users over time.

### 6.4 Design implications

Engaging with speaker hardware can be intimidating to those unfamiliar with the space of speaker design. As designers and roboticists begin to engage with speaker hardware, we encourage them to first outline the major requirements of their system by identifying (1) expected speech or sound profiles that the robot will deploy, (2) loudness requirements and listening angles (if relevant), (3) characteristics of the robot’s I/O and power, (4) any physical engineering requirements for the robot to complete its task (e.g., harsh location, expectations of wear), and (5) the space requirements of the system. These requirements can broadly be used as a guide when determining if a custom speaker should be employed. In the case that a commercial speaker could satisfy design needs, we encourage the reader to select this option; it can save time, be more feasible in the case of mass production, and reduce required expertise. In the case that a custom speaker is justified, for example, via failure to meet one of the above-listed criteria with current hardware, the reader can follow the design steps covered in Section 4. Speaker needs identified at an earlier point in the robot design process can offer advantages such as more flexibility in placement and size, as well as being built into the core hardware of the robot. Space requirements of a given system will directly inform how to maximize internal volume (if custom speakers prove necessary) or select a feasible commercial speaker (if not).

These design considerations are not all-encompassing, but they can be referenced as a general starting point. Other considerations, like the task being performed by the robot, may also have important implications. For example, robots whose primary task involves speaking, like social robots, may be more directly affected by speaker hardware specifics than ones that speak intermittently; future work is necessary to further understand additional contexts and requirements.

## 7 Conclusion

This work sought to push the design space and applications of robot sound forward via speaker analyses, speaker design case studies, and empirical experiments. Results of the work show that fairly simple and low-cost improvements to current robot speakers could be used to retroactively improve the sound quality and communication abilities of robotic platforms in settings from industrial contexts to service. New conventional wisdom for roboticists, such as designing speakers to maximize enclosure volume, quickly incorporating commercial speakers when the application allows it, considering custom speaker design guidelines from this work when robot constraints require, and adding sound to robot behaviors to improve success, can come from this paper. Taken together, our results show that including speakers in robotic systems in some way is more important than picking the perfect speaker. This insight is valuable because many current commercial robots do not include speakers in their design. Further, in more nuanced or challenging environments than the service setting considered in our empirical study, choosing the right type of speaker may have a big impact, as evidenced by our engineering evaluations. For these cases, our results can guide the design and/or selection of the right kind of speaker for the application at hand. As a whole, this work acts to broaden the perspectives of roboticists and designers and enable them to better consider speaker quality in the interactions they create for robots.

## Data Availability

The raw data supporting these conclusions can be requested from the authors upon request with undue reservation.
